# Pharmacokinetic and Pharmacodynamic Profiles of Intravenous and Enteral Nimodipine in Patients with Aneurysmal Subarachnoid Hemorrhage: A Scoping Review

**DOI:** 10.3390/jcm15093425

**Published:** 2026-04-30

**Authors:** Marco Sanvitti, Giada Iafrate, Federico Bilotta

**Affiliations:** 1 Department of Pediatric Surgery, “Sapienza” University of Rome, 00185 Rome, Italy; sanvitti.1927256@studenti.uniroma1.it; 2 Department of Anesthesiology, Intensive Care and Pain Medicine, University of Tor Vergata, 00133 Rome, Italy; giadaiafrate95@libero.it

**Keywords:** aneurysmal subarachnoid hemorrhage, bioavailability, calcium channel blockers, drug administration routes, hemodynamics, nimodipine, pharmacokinetics

## Abstract

**Background**: Nimodipine is routinely used in aneurysmal subarachnoid hemorrhage (aSAH), but the optimal route of administration remains uncertain. Intravenous and enteral delivery differ in pharmacokinetics, yet the clinical relevance of these differences is unclear. This scoping review aimed to map evidence on the pharmacokinetics (PK) and pharmacodynamics (PD) of intravenous and enteral nimodipine and their relationship with clinical outcomes. **Methods**: A scoping review was conducted following PRISMA-ScR guidelines. PubMed, Scopus, and Web of Science were searched from 1982 to March 2026. Studies in adult aSAH patients reporting PK and/or PD outcomes after intravenous or enteral nimodipine were included. Data were synthesized qualitatively. **Results**: Twenty studies were included. Intravenous administration provided higher and more consistent systemic exposure, whereas enteral administration showed low and highly variable bioavailability, particularly via nasogastric tubes. Despite these differences, pharmacodynamic effects were not clearly related to systemic concentrations, and hypotension occurred similarly across routes. Evidence on cerebral physiology was limited. Randomized studies showed no significant differences in delayed cerebral ischemia, infarction, or functional outcomes between routes. **Conclusions**: Pharmacokinetic advantages of intravenous nimodipine do not consistently translate into pharmacodynamic or clinical benefits, although available evidence is limited and heterogeneous. The PK–PD relationship appears weak, and further research is needed to guide optimized administration strategies.

## 1. Introduction

Aneurysmal subarachnoid hemorrhage (aSAH) is a life-threatening cerebrovascular condition associated with high mortality and long-term neurological disability despite advances in neurosurgical and critical care management [1,2]. A key contributor to poor outcomes is delayed cerebral ischemia (DCI), a multifactorial complication involving large-vessel vasospasm, microcirculatory dysfunction, inflammation, and impaired cerebral autoregulation [3]. Preventing DCI remains a central therapeutic goal in the management of aSAH.

Nimodipine, a dihydropyridine calcium channel blocker with high cerebrovascular selectivity, is the only pharmacological agent consistently shown to improve neurological outcomes in patients with aSAH [4]. Its mechanism of benefit is thought to be predominantly neuroprotective, including modulation of intracellular calcium homeostasis and enhancement of microvascular perfusion, rather than solely vasodilation of large cerebral arteries [1,4]. Accordingly, clinical guidelines recommend routine administration of nimodipine in all patients with aSAH [5].

Nimodipine is most commonly administered orally or via enteral feeding tubes, per AHA/ASA guidelines [5]. However, in critically ill patients, factors such as reduced consciousness, impaired gastric motility, and delayed gastric emptying may significantly affect drug absorption [6]. Pharmacokinetic studies have demonstrated substantial variability in plasma concentrations following enteral administration, with some patients exhibiting minimal or negligible drug exposure, particularly in the early phase after hemorrhage or in those with severe neurological injury [7,8].

Intravenous administration offers an alternative route that bypasses gastrointestinal absorption, allowing for more predictable systemic exposure and controlled dosing [9]. Studies have shown that parenteral nimodipine achieves significantly higher area under the concentration–time curve values compared to enteral administration, suggesting greater bioavailability [7,10]. However, intravenous use is associated with an increased risk of systemic hypotension, a critical pharmacodynamic effect that may compromise cerebral perfusion in vulnerable patients [11,12].

Despite these pharmacokinetic differences, clinical outcomes appear comparable between administration routes. A recent network meta-analysis found no significant differences between intravenous and enteral nimodipine in terms of mortality, DCI incidence, or functional outcomes, although both were superior to placebo [13]. This highlights a potential discrepancy between pharmacokinetic parameters and clinical efficacy, and underscores ongoing uncertainty regarding the optimal route of administration.

The aim of this scoping review is to map the available literature on the pharmacokinetics (PK) and pharmacodynamics (PD) of intravenous and enteral nimodipine in patients with aSAH, with the goal of identifying knowledge gaps and informing future research and clinical practice.

## 2. Materials and Methods

This scoping review was conducted in accordance with the Preferred Reporting Items for Systematic Reviews and Meta-analyses for scoping reviews (PRISMA-ScR) guidelines [14]. (PRISMA-ScR Checklist available in Table S1). A protocol for this scoping review was not formally registered.

### 2.1. Search Strategy

The search strategy was based on a combination of keywords from three main conceptual domains: (i) the intervention (nimodipine), (ii) the target condition (subarachnoid hemorrhage, including aneurysmal subarachnoid hemorrhage), and (iii) PK and PD parameters (including drug exposure, absorption, and hemodynamic effects). Dedicated search strings were developed and applied for the following databases: PubMed, Scopus, and Web of Science (complete search strategy is available in Section S1). The search covered studies published from 1 January 1982 to March 2026, with no initial restriction on study design. Database searches were conducted independently by two reviewers (M.S., G.I.), and discrepancies were resolved through discussion with a third reviewer (F.B.).

### 2.2. Risk of Bias and Study Quality Assessment

A formal assessment of study quality or risk of bias was not performed, consistent with the methodological framework of scoping reviews, which aim to map the breadth and nature of available evidence rather than to provide a quantitative or hierarchical evaluation. However, given the heterogeneity of included studies, the absence of structured quality appraisal may limit the interpretability of findings and should be considered when interpreting the results.

### 2.3. Inclusion and Exclusion Criteria

Studies were included if they met the following criteria: (i) published between 1 January 1982 and March 2026; (ii) conducted in human adult populations with aneurysmal subarachnoid hemorrhage; (iii) evaluated nimodipine administered intravenously, orally, or via enteral/transgastric routes; (iv) reported PK and/or PD outcomes (e.g., plasma concentration, AUC, Cmax, Tmax, clearance, bioavailability, or hemodynamic effects such as hypotension). Studies were excluded if they: (i) involved non-human or preclinical models; (ii) were basic science studies without clinical data; (iii) focused exclusively on pediatric or neonatal populations; (iv) did not report PK or PD outcomes; (v) were case reports, small case series, editorials, letters, conference abstracts, narrative reviews, or book chapters unless they provided original pharmacokinetic data relevant to the study objectives; (vi) abstract was not available in English.

### 2.4. Study Selection

All identified records were imported into Rayyan, an online platform designed to support systematic literature screening [15]. After automatic duplicate detection, remaining duplicates were manually removed. Two independent reviewers (M.S., G.I.) screened titles and abstracts in the first phase. Articles deemed potentially eligible were subsequently assessed in full text by two independent reviewers (M.S., F.B.). Disagreements at any stage were resolved by consensus. The final set of included studies was recorded in a structured database. Studies reporting isolated clinical outcomes without pharmacokinetic data were excluded unless they directly compared administration routes or provided clinically relevant pharmacodynamic or safety outcomes.

### 2.5. Data Extraction

Following study selection, data were extracted independently by two reviewers using a standardized data collection form. The following variables were collected for each study: (i) author and year of publication; (ii) study design and sample size; (iii) patient characteristics, including severity of aSAH when available; (iv) route of nimodipine administration (intravenous, oral, enteral, or transgastric); (v) pharmacokinetic parameters (e.g., plasma concentrations, AUC, Cmax, Tmax, clearance, half-life, bioavailability); (vi) pharmacodynamic outcomes, including systemic hemodynamic effects (e.g., hypotension) and, when reported, cerebral perfusion-related parameters; (vii) clinical outcomes related to delayed cerebral ischemia or neurological status, when available.

Studies were subsequently categorized according to predefined domains aligned with the research objectives: (1) PK profile of nimodipine in aSAH; (2) PD effects, including systemic and cerebral hemodynamic impact; (3) comparison between administration routes (intravenous vs. enteral); (4) relationship between drug exposure and clinical outcomes.

## 3. Results

**Figure 1:**
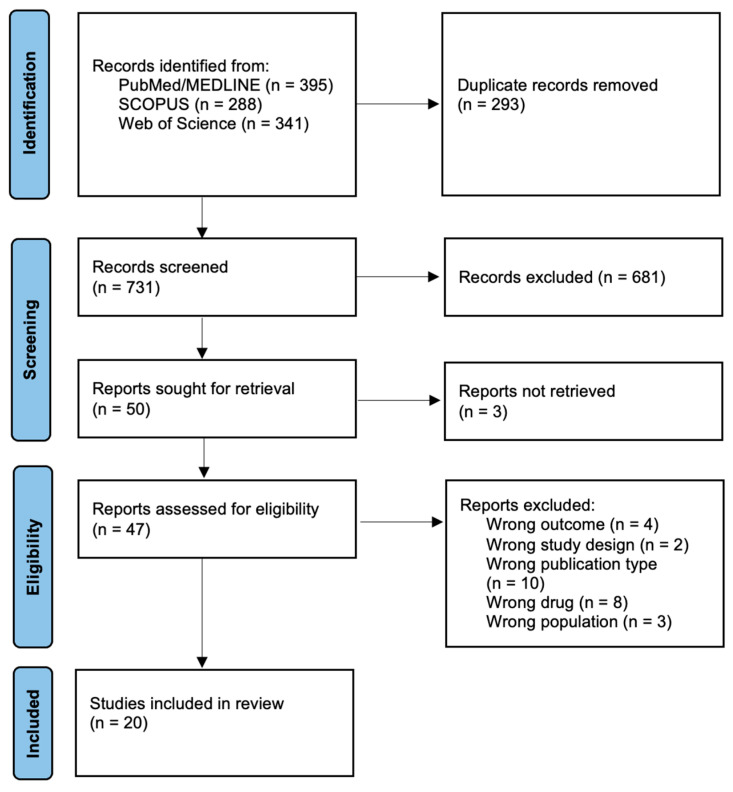
PRISMA flowchart diagram.

Article selection process for this scoping review is represented as a PRISMA Flow Diagram in Figure 1. Following the search strategy, 1024 records were identified. A total of 20 original studies [7,8,10,16,17,18,19,20,21,22,23,24,25,26,27,28,29,30,31,32] were selected for final analysis (Figure 1). Substantial heterogeneity was observed across studies in terms of dosing regimens, administration routes, patient severity, study design, and outcome reporting.

The topics covered are: route of administration, pharmacokinetics, pharmacodynamics, PK–PD relationship, clinical outcomes, administration factors.

### 3.1. Route of Administration

#### 3.1.1. Intravenous Administration

IV nimodipine, typically administered as continuous infusion (1–2 mg/h), was associated with relatively stable plasma concentrations across studies [22,30]. Population PK modeling confirmed moderate interindividual variability (~19%) and identified body surface area as a determinant of clearance, while hemodynamic augmentation did not significantly influence drug exposure [18]. Compared with enteral administration, IV delivery consistently produced more predictable systemic exposure [7,17]

#### 3.1.2. Enteral Administration (Oral and Nasogastric)

Enteral administration demonstrated marked variability in drug exposure. Oral bioavailability ranged widely from approximately 0.6% to 32% across studies [30,31,32] with typical values between 3% and 15% [17,22]. Plasma concentrations following enteral administration were highly variable, and in some cases negligible, particularly in critically ill patients [7,29].

Administration via NG or feeding tubes was consistently associated with reduced exposure compared to oral administration [7,19]. Several studies reported very low or undetectable serum concentrations in patients receiving nimodipine via NG tubes, particularly in those with severe neurological impairment [7,29].

#### 3.1.3. Direct Comparisons Between IV and Enteral Routes

Direct comparisons consistently demonstrated higher systemic exposure during IV administration. AUC values were significantly greater during IV therapy than during oral or NG administration in within-patient analyses [7,29]. Among enteral routes, oral administration resulted in higher exposure than NG administration [7]. One study reported similar systemic concentrations across intravenous, oral, and intra-arterial routes despite marked variability, indicating that differences in exposure may not be consistently observed across all settings [26]. Phase 1 data in healthy volunteers confirmed that IV administration achieved similar overall exposure but with substantially lower variability compared to oral dosing [17]. Across studies, IV administration provided more consistent pharmacokinetic profiles, whereas enteral administration was associated with unpredictable exposure.

### 3.2. Pharmacokinetics

#### 3.2.1. Bioavailability

Oral nimodipine exhibited low and highly variable bioavailability across studies. Reported values ranged from approximately 0.6% to 32% [32], with estimates of 3–10% in early PK studies [22,31] and approximately 7% in controlled crossover data [17]. These findings were consistent across both historical and contemporary ICU-based studies.

#### 3.2.2. Systemic Exposure (AUC and Concentrations)

Systemic exposure varied substantially according to route. IV administration produced stable plasma concentrations and higher AUC values [22,30]. In contrast, enteral administration was associated with lower and highly variable AUC values [7,29]. In some studies, AUC during enteral therapy decreased over time, particularly during the second week after hemorrhage.

#### 3.2.3. Variability

Marked interindividual variability was consistently observed, particularly with enteral administration. Wide ranges in plasma concentrations and AUC values were reported across multiple studies [19,23]. In some patients, especially those receiving NG administration or with severe neurological impairment, drug exposure was minimal or undetectable [7,29].

#### 3.2.4. Factors Influencing Pharmacokinetics

Several factors influencing nimodipine pharmacokinetics were identified. The feeding state significantly affected bioavailability, with reduced exposure under fed conditions [16]. Administration via NG or feeding tubes was consistently associated with reduced exposure compared to oral intake [7,10,19]. Concomitant use of enzyme-inducing drugs such as phenytoin was associated with reduced exposure [19,29]. Patient-related factors, including age and disease severity, also influenced pharmacokinetics [29,30]. Population PK modeling identified body surface area as a determinant of clearance [18].

### 3.3. Pharmacodynamics

#### 3.3.1. Systemic Hemodynamics

Nimodipine administration was associated with systemic hemodynamic effects, particularly reductions in mean arterial pressure. Hypotension was commonly reported and frequently led to dose adjustment or discontinuation [20,24]. However, the relationship between plasma concentrations and hemodynamic effects was not consistently demonstrated across studies. While some investigations reported no significant differences in vasopressor requirements or hemodynamic parameters between IV and enteral administration [21,32], others described measurable reductions in blood pressure and dose-limiting hypotension during treatment [20,23].

#### 3.3.2. Cerebral Physiology

A limited number of studies evaluated cerebral physiological parameters. Nimodipine administration was associated with modest changes in cerebral perfusion pressure and brain tissue oxygenation [20]. In studies assessing cerebrovascular autoregulation, intra-arterial administration produced stronger and more sustained effects compared to IV administration [25]. Overall, cerebral physiological responses were variable and not consistently linked to systemic exposure.

### 3.4. PK–PD Relationship

Only a limited number of studies directly evaluated the relationship between systemic exposure and clinical or physiological effects. Findings were inconsistent. One study reported differential associations between nimodipine enantiomers and outcomes, with distinct relationships between exposure and hemodynamic or vasospasm-related effects [19]. Other studies did not demonstrate a clear association between plasma concentrations and hemodynamic parameters or vasopressor requirements [32]. Overall, evidence linking pharmacokinetics to pharmacodynamic or clinical outcomes was limited.

### 3.5. Clinical Outcomes

Clinical outcomes, including DCI, vasospasm, infarction, functional outcomes, and mortality, were reported in several studies. Randomized controlled trials comparing IV and enteral administration demonstrated no significant differences in DCI incidence, infarction rates, or functional outcomes between routes [27,28]. Observational studies reported inconsistent findings regarding clinical outcomes based on route of administration, with one study showing no significant differences, while others suggested worse outcomes with enteral feeding tube administration [8,10,24].

### 3.6. Administration Factors

Administration-related factors influenced drug exposure and tolerability. NG tube administration and tablet crushing were associated with reduced absorption and lower systemic exposure [7,19]. Feeding conditions affected bioavailability, with reduced exposure observed in fed states [16]. In critically ill patients, impaired gastrointestinal function contributed to variability in drug absorption [29,32].

## 4. Discussion

The aim of this scoping review was to map the PK and PD profiles of intravenous and enteral nimodipine in patients with aSAH. Interpretation of these findings is limited by substantial heterogeneity in study design, dosing regimens, patient populations, and outcome reporting, which precludes direct comparability and quantitative synthesis. This reflects the methodological diversity of the available literature, which limits the applicability of meta-analytic approaches despite the number of studies identified. Intravenous administration provided more predictable and stable systemic exposure, with higher and less variable plasma concentrations, whereas enteral administration, especially via nasogastric tubes, was associated with low and highly variable bioavailability, sometimes resulting in negligible drug levels. Pharmacodynamic effects and clinical outcomes were largely comparable across routes. Hypotension occurred frequently but was not consistently related to drug exposure, and no clear differences were observed in delayed cerebral ischemia or functional outcomes.

The marked variability observed with enteral nimodipine administration is consistent with impaired gastrointestinal absorption in critically ill patients with aSAH [33]. Reduced consciousness, delayed gastric emptying, and intestinal dysmotility are common in this population and can substantially limit drug uptake, particularly in the early phase after hemorrhage. The consistently lower exposure observed with NG administration compared with oral dosing is likely multifactorial, including drug loss during preparation, adsorption to feeding tubes, and altered dissolution [34,35]. The extensive first-pass hepatic metabolism of nimodipine further amplifies the impact of inconsistent absorption on systemic exposure [36]. External factors such as feeding conditions and concomitant use of CYP3A4 inducers, such as phenytoin or anticonvulsants, as well as patient-specific variables including age, disease severity, and body surface area, contribute further to interindividual variability [37,38].

Despite these clear PK differences, PD findings were not consistently aligned with systemic exposure. Differences in PK and clinical findings across studies appear to be influenced by variability in administration route, disease severity, and study design, highlighting the need to interpret results within the context of these factors. Hypotension was the most frequently reported effect, yet its relationship with plasma concentrations remained unclear. Similar hemodynamic profiles and vasopressor requirements were often observed across intravenous and enteral administration, suggesting that higher systemic levels do not necessarily translate into greater systemic effects [21,32]. Several mechanisms may explain this apparent dissociation, including a ceiling effect at the level of vascular smooth muscle, differential activity of nimodipine enantiomers, and a potential mismatch between systemic concentrations and drug activity at the cerebral microcirculatory level [8,39]. Evidence on cerebral physiology was limited and heterogeneous, with only modest and inconsistent effects reported on cerebral perfusion pressure, autoregulation, and brain tissue oxygenation [20,25]. These results suggest that systemic exposure may not reliably predict physiological or clinical response based on currently available evidence.

This apparent discrepancy between pharmacokinetic superiority and clinical outcomes represents the central finding of this review, although it should be interpreted cautiously given the limited number of studies directly evaluating PK–PD relationships and the heterogeneity of reported outcomes. In the reported studies, intravenous administration consistently resulted in higher and more stable systemic exposure [7,29], while randomized studies did not demonstrate differences in delayed cerebral ischemia, infarction, or functional outcomes compared with enteral administration [27,28]. Observational data were similarly inconsistent, with some studies suggesting worse outcomes with NG administration [8,10,24]. These inconsistencies are highly susceptible to confounding by indication, as patients requiring enteral administration via nasogastric or feeding tubes are typically more severely ill. This imbalance in baseline severity is rarely accounted for across studies and may independently influence both pharmacokinetic profiles and clinical outcomes. As a result, differences in outcomes between administration routes cannot be reliably attributed to the route of administration itself. These findings indicate that nimodipine efficacy may not be strictly exposure dependent. A threshold effect may exist, whereby even low or variable concentrations are sufficient to achieve maximal therapeutic benefit. Alternatively, the clinical effect of nimodipine may be primarily mediated through neuroprotective or microcirculatory mechanisms that are not adequately captured by plasma concentrations alone [40,41]. The lack of correlation between systemic exposure and outcomes also suggests that plasma concentrations may be a poor surrogate for drug activity at the site of action within the central nervous system, while CSF fluid may be the more relevant PK parameter. This is supported by recent evidence showing that plasma and CSF concentrations are not consistently correlated and that only low or variably detectable levels are present in brain interstitial fluid, highlighting a potential dissociation between systemic exposure and central drug delivery [42]. Higher CSF nimodipine concentrations and CSF-to-plasma ratios have been associated with better long-term functional outcomes, whereas plasma concentrations showed no such relationship, with no correlation observed between dose or plasma levels and outcome measures [43]. Central exposure may therefore play a more relevant role in mediating therapeutic effects, although the available data remain limited.

From a clinical perspective, these findings support current guideline recommendations favoring enteral administration as the standard approach, given its comparable outcomes and established safety profile. However, the significant variability associated with enteral delivery, particularly via NG tubes, highlights a potential risk of underexposure in critically ill patients. Intravenous administration may therefore represent a useful alternative in selected cases, such as patients with impaired gastrointestinal function or in the early phase after hemorrhage, when absorption is most unreliable. At the same time, the risk of hypotension with intravenous therapy remains a relevant concern and may limit its routine use.

This review also identifies several important knowledge gaps. There is a lack of robust studies directly linking pharmacokinetic parameters to pharmacodynamic effects and clinical outcomes. No therapeutic concentration range for nimodipine has been clearly established, and data on cerebrospinal fluid or brain tissue concentrations remain scarce. The early phase after aSAH, when pharmacokinetic variability appears greatest, is underrepresented in the literature. Furthermore, heterogeneity in dosing regimens, formulations, and administration techniques limits comparability across studies. Future research should focus on direct comparisons between intravenous and enteral administration using standardized protocols and clearly defined PK and PD endpoints. Particular attention should be given to the early phase after hemorrhage, when variability in drug exposure appears greatest. In addition, studies integrating plasma and cerebrospinal fluid measurements are needed to better characterize central nervous system exposure and its relationship with clinical outcomes. Finally, the development of PK–PD models incorporating patient-specific factors may help define individualized dosing strategies and clarify the clinical relevance of systemic versus central drug exposure

The strengths of this review include a comprehensive mapping of PK and PD data across multiple administration routes and study designs, as well as an integrated interpretation of mechanistic and clinical findings. However, several limitations must be acknowledged. The included studies were heterogeneous in design, sample size, and outcome reporting, and the overall number of high-quality randomized trials was limited. As a scoping review, no quantitative synthesis was performed, and conclusions are therefore based on qualitative interpretation of the available evidence. Additionally, data on cerebral physiological endpoints and PK–PD relationships were limited, restricting the ability to draw definitive mechanistic conclusions. The absence of formal critical appraisal limits the ability to assess the internal validity and strength of the included studies, and findings should therefore be interpreted as a descriptive synthesis rather than definitive evidence. The absence of consistent severity stratification further limits the interpretability of comparative outcome data.

## 5. Conclusions

In patients with aneurysmal subarachnoid hemorrhage, intravenous nimodipine provides more predictable systemic exposure, whereas enteral administration is characterized by low and highly variable bioavailability, particularly via nasogastric tubes. However, these pharmacokinetic differences do not consistently translate into pharmacodynamic or clinical advantages, although current evidence remains limited and does not allow definitive conclusions. Systemic exposure appeared to be inconsistently associated with hemodynamic effects and did not predict key outcomes, including delayed cerebral ischemia and functional recovery. Future research should focus on direct comparisons between intravenous and enteral administration using standardized protocols, integrated PK–PD analyses, and evaluation of central nervous system exposure to better define the relationship between route of administration, drug exposure, and clinical outcomes.

## Data Availability

Data sharing is not applicable due to this manuscript does not generate new data.

## Supplementary Materials

The following supporting information can be downloaded at: https://www.mdpi.com/article/10.3390/jcm15093425/s1, Table S1: PRISMA-ScR checklist [14]; Section S1: Search strategy.

## Abbreviations

The following abbreviations are used in this manuscript:

aSAH	Aneurysmal Subarachnoid Hemorrhage
AHA	American Heart Association
ASA	American Stroke Association
AUC	Area Under the Concentration–Time Curve
Cmax	Maximum Plasma Concentration
DCI	Delayed Cerebral Ischemia
ICU	Intensive Care Unit
IV	Intravenous
NG	Nasogastric
PD	Pharmacodynamics
PK	Pharmacokinetics
PK–PD	Pharmacokinetic–Pharmacodynamic Relationship
PRISMA-ScR	Preferred Reporting Items for Systematic Reviews and Meta-Analyses Extension for Scoping Reviews
Tmax	Time to Maximum Plasma Concentration

## References

[1] Connolly E.S., Rabinstein A.A., Carhuapoma J.R., Derdeyn C.P., Dion J., Higashida R.T., Hoh B.L., Kirkness C.J., Naidech A.M., Ogilvy C.S. (2012). Guidelines for the Management of Aneurysmal Subarachnoid Hemorrhage: A Guideline for Healthcare Professionals from the American Heart Association/American Stroke Association. Stroke.

[2] Macdonald R.L., Schweizer T.A. (2017). Spontaneous Subarachnoid Haemorrhage. Lancet.

[3] Vergouwen M.D.I., Vermeulen M., van Gijn J., Rinkel G.J.E., Wijdicks E.F., Muizelaar J.P., Mendelow A.D., Juvela S., Yonas H., Terbrugge K.G. (2010). Definition of Delayed Cerebral Ischemia after Aneurysmal Subarachnoid Hemorrhage as an Outcome Event in Clinical Trials and Observational Studies: Proposal of a Multidisciplinary Research Group. Stroke.

[4] Dorhout Mees S.M., Rinkel G.J.E., Feigin V.L., Algra A., van den Bergh W.M., Vermeulen M., van Gijn J. (2007). Calcium Antagonists for Aneurysmal Subarachnoid Haemorrhage. Cochrane Database Syst. Rev..

[5] Hoh B.L., Ko N.U., Amin-Hanjani S., Chou S.H.-Y., Cruz-Flores S., Dangayach N.S., Derdeyn C.P., Du R., Hänggi D., Hetts S.W. (2023). 2023 Guideline for the Management of Patients with Aneurysmal Subarachnoid Hemorrhage: A Guideline From the American Heart Association/American Stroke Association. Stroke.

[6] MacLaren R. (2023). Considerations When Administering Medications Enterally in the Critically Ill. Curr. Opin. Clin. Nutr. Metab. Care.

[7] Abboud T., Andresen H., Koeppen J., Czorlich P., Duehrsen L., Stenzig J., Westphal M., Regelsberger J. (2015). Serum Levels of Nimodipine in Enteral and Parenteral Administration in Patients with Aneurysmal Subarachnoid Hemorrhage. Acta Neurochir..

[8] Isse F.A., Abdallah Y.E.H., Mahmoud S.H. (2020). The Impact of Nimodipine Administration through Feeding Tube on Outcomes in Patients with Aneurysmal Subarachnoid Hemorrhage. J. Pharm. Pharm. Sci..

[9] Morales Castro D., Dresser L., Granton J., Fan E. (2023). Pharmacokinetic Alterations Associated with Critical Illness. Clin. Pharmacokinet..

[10] Mahmoud S.H., Hefny F.R., Panos N.G., Delucilla L., Ngan Z., Perreault M.M., Hamilton L.A., Rowe A.S., Buschur P.L., Owusu-Guha J. (2023). Comparison of Nimodipine Formulations and Administration Techniques via Enteral Feeding Tubes in Patients with Aneurysmal Subarachnoid Hemorrhage: A Multicenter Retrospective Cohort Study. Pharmacotherapy.

[11] Hänggi D., Etminan N., Aldrich F., Steiger H.J., Mayer S.A., Diringer M.N., Hoh B.L., Mocco J., Faleck H.J., Macdonald R.L. (2017). Randomized, Open-Label, Phase 1/2a Study to Determine the Maximum Tolerated Dose of Intraventricular Sustained Release Nimodipine for Subarachnoid Hemorrhage (NEWTON [Nimodipine Microparticles to Enhance Recovery While Reducing Toxicity After Subarachnoid Hemorrhage]). Stroke.

[12] Rass V., Kindl P., Lindner A., Kofler M., Altmann K., Putnina L., Ianosi B.-A., Schiefecker A.J., Beer R., Pfausler B. (2023). Blood Pressure Changes in Association with Nimodipine Therapy in Patients with Spontaneous Subarachnoid Hemorrhage. Neurocrit. Care.

[13] Lei G., Rao Z., Hu Y. (2023). The Efficacy of Different Nimodipine Administration Route for Treating Subarachnoid Hemorrhage: A Network Meta-Analysis. Medicine.

[14] Tricco A.C., Lillie E., Zarin W., O’Brien K.K., Colquhoun H., Levac D., Moher D., Peters M.D.J., Horsley T., Weeks L. (2018). PRISMA Extension for Scoping Reviews (PRISMA-ScR): Checklist and Explanation. Ann. Intern. Med..

[15] Ouzzani M., Hammady H., Fedorowicz Z., Elmagarmid A. (2016). Rayyan-a Web and Mobile App for Systematic Reviews. Syst. Rev..

[16] Bellapart J., Hernández-Mitre M.P., Wu X., Wallis S.C., Smith M.-L., Stuart J., Fourie C., Livermore A., Roberts J.A. (2026). Nimodipine Dosing and Pharmacokinetic Variability in Subarachnoid Hemorrhage Patients. J. Clin. Neurosci..

[17] Kumar A., D’Andrea C., Kohli P., Kottayil S.G., Longstreth J., Macdonald R.L. (2025). Phase 1, Randomized, Crossover Study Comparing Intravenous GTX-104 to Oral Nimodipine in Healthy Human Subjects. PLoS ONE.

[18] Foucher A., Gregoire M., Dauvergne J.E., Boissier E., Rozec B., Deslandes G., Lakhal K. (2025). Impact of Cerebral Vasospasm Therapy on Nimodipine Exposure in Subarachnoid Hemorrhage: A Population Pharmacokinetic Study. J. Neurol. Sci..

[19] Mahmoud S.H., Hefny F., Isse F.A., Farooq S., Ling S., O’Kelly C., Kutsogiannis D.J. (2024). Nimodipine Systemic Exposure and Outcomes Following Aneurysmal Subarachnoid Hemorrhage: A Pilot Prospective Observational Study (ASH-1 Study). Front. Neurol..

[20] Moser M., Schwarz Y., Herta J., Plöchl W., Reinprecht A., Zeitlinger M., Brugger J., Ramazanova D., Rössler K., Hosmann A. (2024). The Effect of Oral Nimodipine on Cerebral Metabolism and Hemodynamic Parameters in Patients Suffering Aneurysmal Subarachnoid Hemorrhage. J. Neurosurg. Anesthesiol..

[21] Göttsche J., Schweingruber N., Groth J.C., Gerloff C., Westphal M., Czorlich P. (2021). Safety and Clinical Effects of Switching From Intravenous to Oral Nimodipine Administration in Aneurysmal Subarachnoid Hemorrhage. Front. Neurol..

[22] Kumana C.R., Kou M., Yu Y.L., Fong K.Y., Fung C.F., Chang C.M., Mück W., Lauder I.J. (1993). Investigation of Nimodipine Pharmacokinetics in Chinese Patients with Acute Subarachnoid Haemorrhage. Eur. J. Clin. Pharmacol..

[23] Kieninger M., Gruber M., Knott I., Dettmer K., Oefner P.J., Bele S., Wendl C., Tuemmler S., Graf B., Eissnert C. (2019). Incidence of Arterial Hypotension in Patients Receiving Peroral or Continuous Intra-Arterial Nimodipine After Aneurysmal or Perimesencephalic Subarachnoid Hemorrhage. Neurocrit. Care.

[24] Samseethong T., Suansanae T., Veerasarn K., Liengudom A., Suthisisang C. (2018). Impact of Early Versus Late Intravenous Followed by Oral Nimodipine Treatment on the Occurrence of Delayed Cerebral Ischemia Among Patients with Aneurysm Subarachnoid Hemorrhage. Ann. Pharmacother..

[25] Hockel K., Diedler J., Steiner J., Birkenhauer U., Ernemann U., Schuhmann M.U. (2017). Effect of Intra-Arterial and Intravenous Nimodipine Therapy of Cerebral Vasospasm After Subarachnoid Hemorrhage on Cerebrovascular Reactivity and Oxygenation. World Neurosurg..

[26] Albanna W., Weiss M., Conzen C., Clusmann H., Schneider T., Reinsch M., Müller M., Wiesmann M., Höllig A., Schubert G.A. (2017). Systemic and Cerebral Concentration of Nimodipine During Established and Experimental Vasospasm Treatment. World Neurosurg..

[27] Soppi V., Karamanakos P.N., Koivisto T., Kurki M.I., Vanninen R., Jaaskelainen J.E., Rinne J. (2012). A Randomized Outcome Study of Enteral versus Intravenous Nimodipine in 171 Patients After Acute Aneurysmal Subarachnoid Hemorrhage. World Neurosurg..

[28] Kronvall E., Undrén P., Romner B., Säveland H., Cronqvist M., Nilsson O.G. (2009). Nimodipine in Aneurysmal Subarachnoid Hemorrhage: A Randomized Study of Intravenous or Peroral Administration. J. Neurosurg..

[29] Soppi V., Kokki H., Koivisto T., Lehtonen M., Helin-Tanninen M., Lehtola S., Rinne J. (2007). Early-Phase Pharmacokinetics of Enteral and Parenteral Nimodipine in Patients with Acute Subarachnoid Haemorrhage—A Pilot Study. Eur. J. Clin. Pharmacol..

[30] Vinge E., Andersson K.E., Brandt L., Ljunggren B., Nilsson L.G., Rosendal-Helgesen S. (1986). Pharmacokinetics of Nimodipine in Patients with Aneurysmal Subarachnoid Haemorrhage. Eur. J. Clin. Pharmacol..

[31] Rämsch K.D., Ahr G., Tettenborn D., Auer L.M. (1985). Overview on Pharmacokinetics of Nimodipine in Healthy Volunteers and in Patients with Subarachnoid Hemorrhage. Minim. Invasive Neurosurg..

[32] Sanders E.L., Wilhelm A., Kors B.M., Girbes A., Swart E. (2011). Pharmacokinetics of Intravenous and Oral Nimodipine in ICU Patients with a Subarachnoid Haemorrhage. Pharm. Weekbl..

[33] Zou T., He H., Wu Y., Xie X., Yin W. (2025). Gastrointestinal Dysfunction in Aneurysmal Subarachnoid Hemorrhage: Prevalence, Clinical Correlates, and Prognostic Implications from a 15-Year ICU Cohort Study. Front. Neurol..

[34] Ruzsíková A., Součková L., Suk P., Opatřilová R., Kejdušová M., Šrámek V. (2015). Quantitative Analysis of Drug Losses Administered via Nasogastric Tube--In Vitro Study. Int. J. Pharm..

[35] Zhu L.-L., Zhou Q. (2013). Therapeutic Concerns When Oral Medications Are Administered Nasogastrically. J. Clin. Pharm. Ther..

[36] Clough B., Tenii J., Wee C., Gunter E., Griffin T., Aiyagari V. (2022). Nimodipine in Clinical Practice: A Pharmacological Update. J. Neurosci. Nurs..

[37] Mück W., Breuel H.P., Kuhlmann J. (1996). The Influence of Age on the Pharmacokinetics of Nimodipine. Int. J. Clin. Pharmacol. Ther..

[38] Tartara A., Galimberti C.A., Manni R., Parietti L., Zucca C., Baasch H., Caresia L., Mück W., Barzaghi N., Gatti G. (1991). Differential Effects of Valproic Acid and Enzyme-Inducing Anticonvulsants on Nimodipine Pharmacokinetics in Epileptic Patients. Br. J. Clin. Pharmacol..

[39] Scriabine A., van den Kerckhoff W. (1988). Pharmacology of Nimodipine. A Review. Ann. N. Y. Acad. Sci..

[40] Frank R., Szarvas P.A., Pesti I., Zsigmond A., Berkecz R., Menyhárt Á., Bari F., Farkas E. (2024). Nimodipine Inhibits Spreading Depolarization, Ischemic Injury, and Neuroinflammation in Mouse Live Brain Slice Preparations. Eur. J. Pharmacol..

[41] Pesti I., Varga V., Qorri E., Frank R., Kata D., Vinga K., Szarvas P.A., Menyhárt Á., Gulya K., Bari F. (2025). Nimodipine Reduces Microglial Activation in Vitro as Evidenced by Morphological Phenotype, Phagocytic Activity and High-Throughput RNA Sequencing. Br. J. Pharmacol..

[42] Moser M.M., Rössler K., Hirschmann D., Gramss L., Tahir A., Plöchl W., Herta J., Reinprecht A., Zeitlinger M., Hosmann A. (2025). Cerebral Ischemia Protection After Aneurysmal Subarachnoid Hemorrhage: CSF Nimodipine Levels After Intravenous Versus Oral Nimodipine Administration. Clin. Pharmacol. Ther..

[43] Riva R., Pegoli M., Contin M., Perrone A., Mohamed S., Zanello M. (2019). Cerebrospinal Fluid Concentrations of Nimodipine Correlate with Long-Term Outcome in Aneurysmal Subarachnoid Hemorrhage: Pilot Study. Clin. Neuropharmacol..

